# Fermented Corn–Soybean Meal Improved Growth Performance and Reduced Diarrhea Incidence by Modulating Intestinal Barrier Function and Gut Microbiota in Weaned Piglets

**DOI:** 10.3390/ijms25063199

**Published:** 2024-03-11

**Authors:** Yueqin Qiu, Jiaxi Tang, Li Wang, Xuefen Yang, Zongyong Jiang

**Affiliations:** 1 State Key Laboratory of Swine and Poultry Breeding Industry, Guangzhou 510640, China; tangjiaxi@gdaas.cn (J.T.); wangli1@gdaas.cn (L.W.); jiangzongyong@gdaas.cn (Z.J.); 2 Key Laboratory of Animal Nutrition and Feed Science in South China, Ministry of Agriculture and Rural Affairs, Guangzhou 510640, China; 3 Guangdong Provincial Key Laboratory of Animal Breeding and Nutrition, Guangzhou 510640, China; 4 Institute of Animal Science, Guangdong Academy of Agricultural Sciences, Guangzhou 510640, China

**Keywords:** fermented mixed feed, weaned piglets, growth performance, intestinal barrier, butyrate, gut microbiota

## Abstract

This study aimed to investigate the effects of fermented corn–soybean meal mixed feed (FMF) on growth performance, intestinal barrier function, gut microbiota and short-chain fatty acids in weaned piglets. A total of 128 weaned piglets [Duroc×(Landrace×Yorkshire), male, 21-day-old] were randomly allocated to four groups. Piglets were fed a control diet (CON) or the control diet supplemented with 10%, 50% or 100% FMF (FMF-10, FMF-50 or FMF-100, respectively) for 14 d. The results showed that the FMF-100 group had higher average daily gain and average daily feed intake and lower diarrhea incidence than the CON group (*p <* 0.05). The FMF-50 and FMF-100 groups had greater villus height in the duodenum and jejunum, and the FMF-10 and FMF-100 groups had higher villus height-to-crypt depth ratio in the duodenum and jejunum than the CON group. Additionally, the FMF-100 group had higher protein expression of duodenal, jejunal and ileal ZO-1 and jejunal claudin-1; higher mRNA expression of duodenal and ileal *TJP1* and jejunal *CLDN1* and *IL10*; and lower jejunal *IL1B* mRNA expression (*p <* 0.05). The FMF-50 group showed higher jejunal ZO-1 and claudin-1 protein levels, higher mRNA expression levels of *IL10* and *TJP1* and lower levels of *TNF* in the jejunum; the FMF-10 group had higher mRNA expression levels of *IL10* and lower levels of *TNF* in the jejunum than the CON group (*p <* 0.05). Furthermore, the FMF-10 and FMF-50 groups had higher colonic *Lactobacillus* abundance and butyrate levels; the FMF-100 group had higher abundance of colonic butyrate, *Lactobacillus* and *Faecalibacterium* than the CON group (*p <* 0.05). Collectively, our results suggest that FMF could improve intestinal mucosal barrier function, gut microbiota and their metabolites, thereby enhancing average daily gain and reducing diarrhea incidence in weaned piglets.

## 1. Introduction

Weaning stress often impairs intestinal barrier function, including destruction of tight junctions (TJs), secretion of inflammatory factors and imbalance of gut microbiota, which ultimately leads to an increase in diarrhea incidence and a reduction in growth performance in weaned piglets [1,2,3]. In the last two decades, fermented feed has been regarded as an effective alternative to antibiotic growth and has been promoted in swine production. Microbial fermentation using bacteria can degrade anti-nutritional compounds, undigested components and some large-size nutrients in feed while providing probiotics and metabolites [4,5,6,7]. Additionally, fermented feed has been shown to improve intestinal morphology [8] and gut microbiota [9,10], decrease diarrhea incidence [11] and thus benefit growth performance [10,12]. Corn and soybean meal are the major feed ingredients in pig feed. However, these feedstuffs contain high levels of anti-nutritional factors, such as soybean antigenic proteins and phytic acid, which reduce diet nutrient digestibility and have negative health effects on piglets [13]. It has been reported that fermented corn–soybean meal mixed feed (FMF) with *Bacillus subtilis* and *Enterococcus faecium* produces a large amount of lactic acid; breaks down larger soy proteins, including β-conglycininα’-subunit (76 kDa), to lengths of 35 kDa and under; reduces soybean antigenic proteins (β-conglycinin and glycinin); and increases the concentration of crude protein [4]. Previous studies have demonstrated that fermented soybean meal improves growth performance, intestinal morphology, nutrient digestibility and gut microbiota in weaned piglets [10,12,14,15]. However, these studies have not investigated the effects of fermented soybean meal on intestinal tight junctions and intestinal inflammation in weaned piglets. Additionally, although previous studies have demonstrated that fermented corn–soybean meal feed increases serum immunity, regulates mRNA abundance of antimicrobial peptides and Toll-like receptors and regulates gut microbiota in grower-finisher pigs [16,17], the effects of FMF in weaned piglets need further exploration.

This study was conducted to investigate the effects of fermented corn–soybean meal mixed feed on growth performance, nutrient digestibility, intestinal barrier function, gut microbiota and short-chain fatty acids in weaned piglets. We hypothesized that fermented corn–soybean meal feed would have positive effects on growth performance and intestinal health in weaned piglets.

## 2. Results

### 2.1. Analysis of Substrates before and after Fermentation

**Figure 1:**
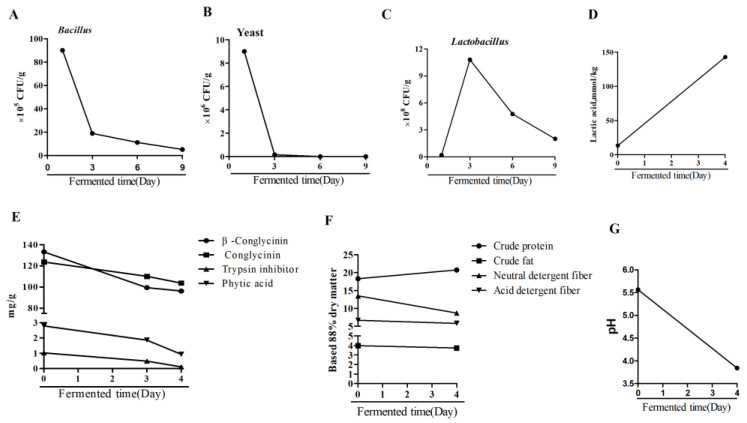
Changes in microbial composition, lactic acid concentration, pH and chemical composition of diets before and after fermentation. During fermentation, changes in the quantities of (**A**) *Bacillus*, (**B**) yeast and (**C**) *Lactobacillus* on days 1, 3, 6 and 9. (**D**) Lactic acid concentration on days 0 and 4. (**E**) Anti-nutritional factors levels on days 1, 2 and 4. (**F**) Chemical composition on days 0 and 4. (**G**) pH values on days 0 and 4.

As shown in Figure 1, when fermentation was prolonged, there was a large increase in the quantity of *Lactobacillus* in the fermented substrates on day 3; however, this quantity decreased during the subsequent fermentation period. Additionally, the quantities of *Bacillus* and yeast gradually decreased with the increase in fermentation time, and the final count was below the level of detection for yeast in the fermented substrates on day 9. After fermentation, the levels of anti-nutritional factors including β-conglycinin, trypsin inhibitor and phytic acid were decreased, and the lactic acid level was increased. Compared with the non-fermented substrates, the fermented substrates had a higher level of crude protein and a lower level of neutral detergent fiber. Additionally, the pH was decreased after fermentation.

### 2.2. Effects of Fermented Mixed Feed on Growth Performance and Nutrient Digestibility in Weaned Piglets

**Figure 2:**
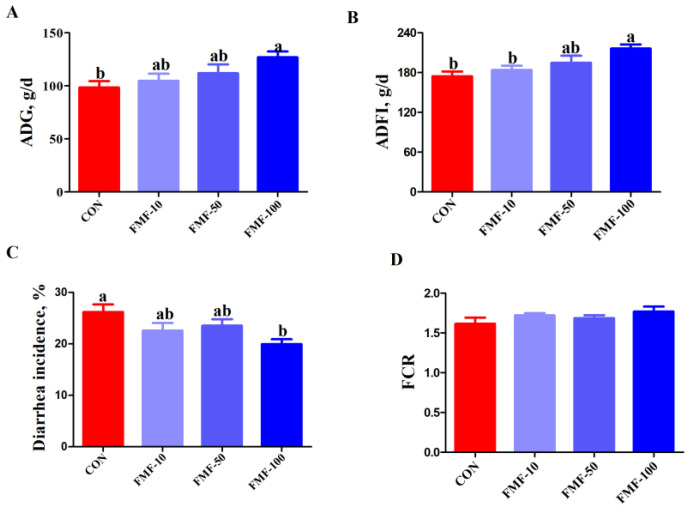
Effects of fermented mixed feed supplementation on growth performance in weaned piglets. (**A**) ADG; (**B**) ADFI; (**C**) diarrhea incidence; (**D**) feed-to-gain ratio among the different dietary groups. Values are means ± SEMs; *n* = 8 per treatment. Error bars represent 95% confidence intervals, ^a,b^ indicate that means with different letters were statistically significant among treatment groups (*p* < 0.05). ADG, average daily gain; ADFI, average daily feed intake; FCR, feed conversion ratio; CON, control diet; FMF-10, FMF-50 and FMF-100, control diet supplemented with 10%, 50% and 100% fermented corn–soybean meal mixed feed (FMF), respectively.

As shown in Figure 2, the piglets in the FMF-100 group had higher average daily gain (ADG) and average daily feed intake (ADFI) values (calculated based on wet weight and dry matter) (*p <* 0.05) and significantly lower diarrhea incidence than those in the CON group (*p <* 0.05). In addition, there were no significant differences in the feed conversion ratio (FCR) among the treatments. The digestibility of crude protein and crude fiber in piglets in the FMF-100 group was significantly increased (*p* < 0.05) compared with the CON group (Table 1).

### 2.3. Effect of Fermented Mixed Feed on Intestinal Morphology in Weaned Piglets

**Figure 3:**
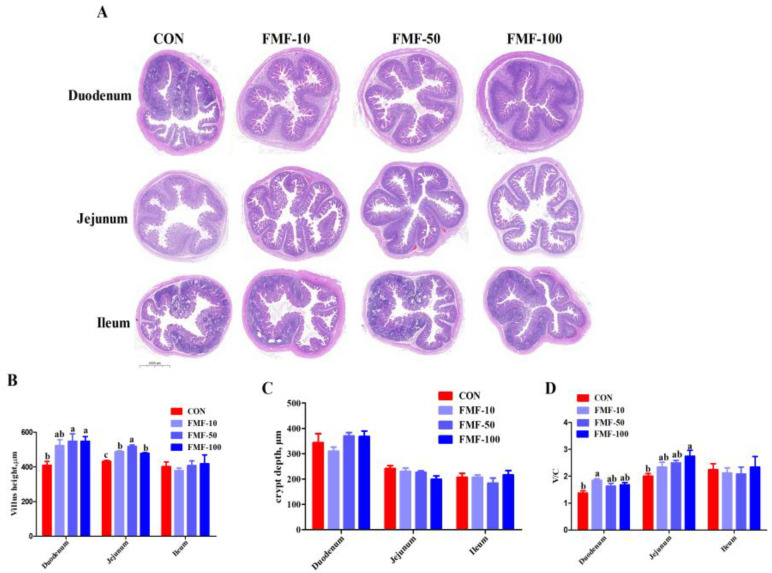
Effects of fermented mixed feed supplementation on intestinal morphology in weaned piglets. (**A**) Hematoxylin–eosin (HE)-stained images of duodenum, jejunum and ileum. Bar chart showing (**B**) villus height, (**C**) crypt depth and (**D**) V/C ratio in the duodenum, jejunum and ileum. Values are means ± SEMs; *n* = 8 per treatment. Error bars represent 95% confidence intervals; ^a,b,c^ indicate that means with different letters were statistically significant among treatment groups (*p* < 0.05). CON, control diet; FMF-10, FMF-50 and FMF-100, control diet supplemented with 10%, 50% and 100% fermented corn–soybean meal mixed feed (FMF), respectively; V/C, villus height-to-crypt depth ratio.

The histological analysis showed that piglets in the FMF-50 and FMF-100 groups presented greater villus height in the duodenum than those in the CON group (*p <* 0.05). Compared with the control diet, 50% and 100% fermented corn–soybean meal feed supplementation significantly increased villus height in the duodenum (*p <* 0.05), while 10%, 50% and 100% fermented corn–soybean meal feed supplementation significantly increased villus height in the jejunum (*p <* 0.05). Furthermore, 10% fermented corn–soybean meal feed significantly increased the villus height-to-crypt depth (V/C) ratio in the duodenum (*p <* 0.05), and 100% fermented corn–soybean meal feed significantly increased the V/C ratio in the jejunum (*p <* 0.05) compared with the control diet (Figure 3). Additionally, there were no significant differences in villus height and V/C ratio in the ileum nor in intestinal crypt depth among the treatments.

### 2.4. Effects of Fermented Mixed Feed on Expression of Genes Related to Tight Junction Proteins and Inflammation in Weaned Piglets

**Figure 4:**
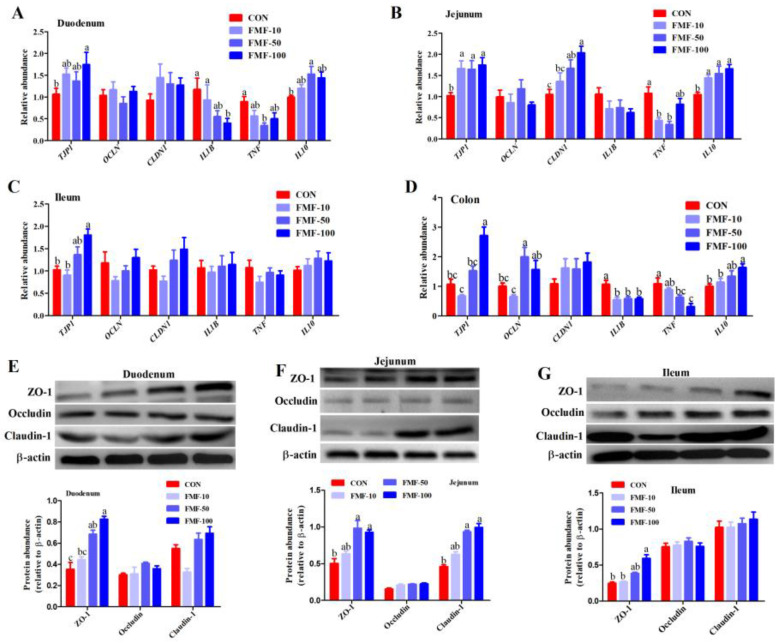
Effects of fermented mixed feed supplementation on the expression of tight junction proteins and cytokines in the duodenum, jejunum, ileum and colon mucosa of weaned piglets. Relative mRNA expression levels of tight junctions proteins and cytokines (**A**) in the duodenum, (**B**) in the jejunum, (**C**) in the ileum and (**D**) in the colon; protein expression levels of ZO-1, claudin-1 and occludin, and the ratio of ZO-1, claudin-1 and occludin to β-actin (**E**) in the duodenum, (**F**) in the jejunum and (**G**) in the ileum. Values are means ± SEMs; *n* = 8 per treatment. Error bars represent 95% confidence intervals; ^a,b,c^ indicate that means with different letters were statistically significant among treatment groups (*p* < 0.05). CON, control diet; FMF-10, FMF-50 and FMF-100, control diet supplemented with 10%, 50% and 100% fermented corn–soybean meal mixed feed (FMF), respectively; IL1B, Interleukin 1 beta; IL10, interleukin 10; TNF, tumor necrosis factor alpha; TJP1, tight junction protein 1; ZO-1, zonula occludens-1.

As shown in Figure 4, in the duodenum, compared with piglets in the CON group, piglets in the FMF-100 groups presented higher mRNA expression levels of *TJP1* (fold change/FC = 1.74 for FMF-100 vs. CON) and lower expression levels of *IL1B* (FC = 0.40 for FMF-100 vs. CON), and piglets in the FMF-50 group presented higher mRNA expression levels of *IL10* (FC = 1.53 for FMF-50 vs. CON) and lower mRNA expression levels of *TNF* (FC = 0.35 for FMF-50 vs. CON) (*p <* 0.05). In the jejunum, compared with piglets in the CON group, piglets in the FMF-10 group presented higher expression levels of *TJP1* (FC = 1.93 for FMF-10 vs. CON) and *IL10* transcripts and lower mRNA expression levels of *TNF* (FC = 0.43 for FMF-10 vs. CON); piglets in the FMF-50 group presented higher expression levels of *TJP1* (FC = 1.88 for FMF-50 vs. CON) and *IL10* (FC = 1.55 for FMF-50 vs. CON) transcripts and lower mRNA expression levels of *TNF* (FC = 0.33 for FMF-50 vs. CON); piglets in the FMF-100 group showed higher mRNA expression levels of *CLDN1* (FC = 2.04 for FMF-100 vs. CON) and *IL10* (FC = 1.66 for FMF-100 vs. CON) (*p <* 0.05). In the ileum, piglets in the FMF-100 group showed higher mRNA expression levels of *TJP1* (FC = 1.80 for FMF-100 vs. CON) than those in the CON group levels (*p <* 0.05). Piglets in the FMF-100 group showed higher mRNA expression levels of colonic *TJP1* (FC = 3.26 for FMF-100 vs. CON) and *IL10* (FC = 1.63 for FMF-100 vs. CON) transcripts than those in the CON group and FMF-10 group (*p <* 0.05). Additionally, 10%, 50% and 100% fermented corn–soybean meal feed significantly decreased mRNA expression levels of colonic *IL1B* (*p <* 0.05).

At the protein level, piglets in the FMF-100 group presented higher duodenal ZO-1 protein expression levels than those in the CON group (*p <* 0.05) (Figure 4E); further, 50% and 100% fermented corn–soybean meal feed significantly increased the protein expression levels of jejunal ZO-1 and claudin-1 compared with the control diet (*p <* 0.05) (Figure 4F); piglets in the FMF-100 group presented higher ileal claudin-1 protein expression levels than those in the CON group (*p <* 0.05) (Figure 4G). Additionally, there were no significant differences in the occludin protein expression levels in the duodenum, jejunum and ileum nor in the claudin-1 protein expression levels in the duodenum and ileum among the treatments.

### 2.5. Effects of Fermented Mixed Feed on the Short-Chain Fatty acid Levels in the Colon of Weaned Piglets

**Figure 5:**
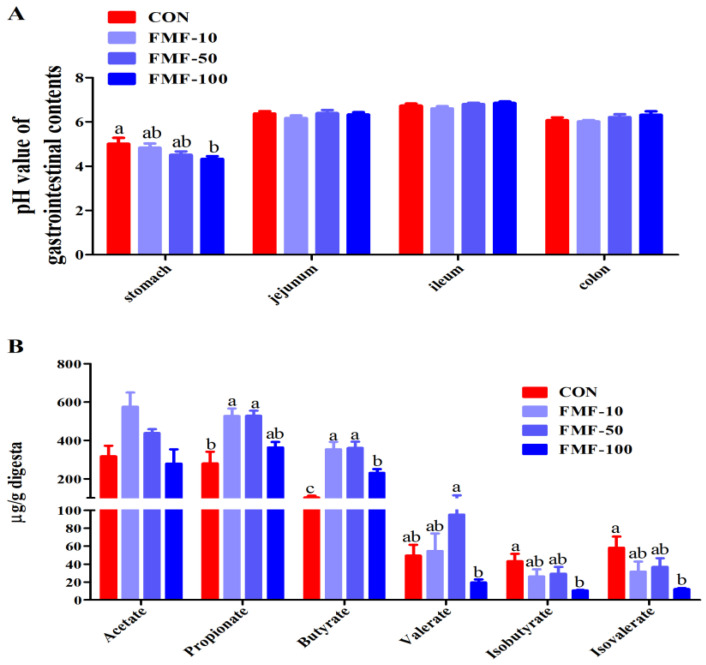
Effect of fermented mixed feed supplementation on short-chain fatty acids (SCFAs) in the colonic contents. (**A**) Determination of pH values of the stomach, jejunum, ileum and colon contents. (**B**) The levels of SCFAs, including acetic acid, propionic acid, butyric acid, valeric acid, isovaleric acid and isobutyric acid, in the colonic contents. Values are means ± SEMs; *n* = 8 per treatment. Error bars represent 95% confidence intervals; ^a,b,c^ indicate that means with different letters were statistically significant among treatment groups (*p* < 0.05). CON, control diet; FMF-10, FMF-50 and FMF-100, control diet supplemented with 10%, 50% and 100% fermented corn–soybean meal mixed feed (FMF), respectively; SCFAs, short-chain fatty acids.

The results showed that the stomach contents of piglets in the FMF-100 group had a lower pH value compared with the CON group (*p <* 0.05), and there were no significant differences in the pH values of the jejunal, ileal and colonic contents among treatments (Figure 5A). The colonic levels of propionate and butyrate (FC = 3.46 and 3.53 for FMF-10 vs. CON and FMF-50 vs. CON, respectively) were significantly higher in the FMF-10 and FMF-50 groups than in the CON group (*p <* 0.05) (Figure 5B). Moreover, the butyrate levels in the FMF-100 group were increased (FC = 2.25 for FMF-100 vs. CON), and the levels of branched-chain fatty acids (BCFAs) including isobutyrate and isovelarate were decreased compared with those in the CON group (*p <* 0.05) (Figure 5B). Moreover, fermented corn–soybean meal feed had no effects on the acetate levels (Figure 5B).

### 2.6. Effects of Fermented Mixed Feed on the Composition of the Colonic Microbiota in Weaned Piglets

**Figure 6:**
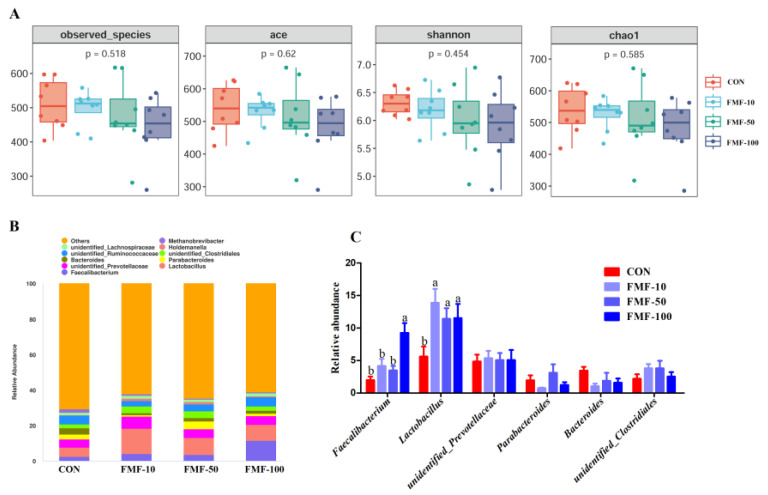
Effects of fermented mixed feed supplementation on alpha and beta diversity and microbial composition. (**A**) Bacterial α diversity indices. (**B**) Stacked bar chart showing the relative abundance of colonic bacteria at the genus level (top 10) in the different treatment groups. (**C**) Bar chart showing significantly altered bacteria at the genus level. Values are means ± SEMs; *n* = 8 per treatment. Error bars represent 95% confidence intervals; ^a,b^ indicate that means with different letters were statistically significant among treatment groups (*p* < 0.05). CON, control diet; FMF-10, FMF-50 and FMF-100, control diet supplemented with 10%, 50% and 100% fermented corn–soybean meal mixed feed (FMF), respectively.

The alpha diversity of colonic microbiota indicated by ace, observed species, shan and chao1 is shown in Figure 6A. There were no significant differences in these indicators among treatments (*p* > 0.05). As shown in Figure 6B,C, at the genus level, piglets in the FMF-100 group showed a significant increase in the relative abundance of *Faecalibacterium* (*p <* 0.05) compared with those in the CON, FMF-10 and FMF-50 groups. The relative abundance of *Lactobacillus* was higher in the FMF-10, FMF-50 and FMF-100 groups than in the CON group.

## 3. Discussion

Fermented feed in pig diet has received attention due to its potential effects on growth performance and gut health in weaned piglets. As expected, our results showed that fermented corn–soybean meal complete replacement of the corresponding un-fermented substrates in the control diet significantly increased ADG and ADFI in weaned piglets, which is consistent with the results of several previous studies investigating the effects of fermented feed [15,18,19]. Obviously, increases in body weight and ADFI in the FMF-100 group might be partly attributed to the increase in crude protein content and the elimination of β-conglycinin, trypsin inhibitor and phytic acid in fermented mixed feed, which has a potential capability to improve nutrient apparent total-tract digestibility and growth efficiency in weaned piglets [20,21]. Consistently, the results in the present study showed that the digestibility of crude protein and crude fiber in weaned piglets in the FMF-100 group was higher than in the CON group.

Moreover, we also found that 100% fermented corn–soybean meal replacement significantly decreased diarrhea incidence in the present study. This finding is consistent with previous studies, which reported that fermented feed reduced post-weaning diarrhea [14,15,22]. A possible reason could be that the increase in lactic acid level and reduction in pH value after fermentation as observed in this study may contribute to inhibiting the growth of pathogenic bacteria and consequently alleviate diarrhea [23]. Additionally, the increased *Lactobacillus* abundance and the dead cells of *Bacillus* and yeast in fermented feed in this study might probiotically or prebiotically affect intestinal barrier function and digestive tract microflora, which is beneficial to reducing diarrhea [10,24]

Villus height, crypt depth and V/C are mainly used to evaluate intestinal morphology and functions [25]. In the present study, fermented mixed feed and especially entire FMF replacement increased villus height in the duodenum and jejunum and jejunal V/C, suggesting that fermented mixed feed improved the intestinal morphology. Consistently with our results, Zhu et al. showed that fermented soybean meal increased villus height and decreased crypt depth in the duodenum, jejunum and ileum [12]. Similarly, a study by Xie et al. reported that fermented soybean meal induced an increase in jejunal villus height and V/C [15]. Correspondingly, the results of the present study showed that fermented corn–soybean meal feed also significantly upregulated the expression of *TJP1* and *CLDN1* mRNA and their proteins. These tight junction proteins are major integral membrane proteins that can limit epithelial permeability to low-molecular-mass molecules and maintain barrier function [26]. Studies have reported that weaning stress induces susceptibility of the gastrointestinal trait to *Escherichia coli* invasion, which stimulates the intestinal mucosa to produce inflammatory factors and injures intestinal barrier function [3,27]. Su et al. found that fermented defatted rice bran reduced serum pro-inflammatory cytokines including TNF-α, IL-1β and INF-γ in finishing pigs [28]. Consistently with these observations, we found that fermented corn–soybean meal feed significantly decreased the mRNA expression levels of pro-inflammatory cytokines such as *TNF* and *IL-1B* and enhanced *IL10* gene expression levels in the small intestine and colon. Thus, the fact that fermented corn–soybean meal feed protected weaned piglets from intestinal inflammatory response might be linked to enhanced intestinal barrier function. Meanwhile, the modulation of gut microbiota and their metabolites by fermented corn–soybean meal also contributed to alleviating intestinal inflammation [29].

Previous studies have reported that propionate and butyrate exert anti-inflammatory effects and modulate intestinal barrier function [30,31]. In the present study, our results show that 10% and 50% fermented corn–soybean meal feed increased the colonic levels of propionate and butyrate and that 100% fermented corn–soybean meal feed increased the colonic butyrate levels, which suggests that the increase in butyrate and propionate by fermented corn–soybean meal might be linked to the alleviation of intestinal inflammation. The results of the present study also reveal that 100% fermented corn–soybean meal decreased BCFAs including isobutyrate and isovalerate, which are involved in nitrogen metabolism [32]. This finding is in line with the result that the digestibility of crude protein was increased after fermentation. However, unlike the well-studied acetate, butyrate and propionate, isobutyrate and isovalerate produced by the gut microbiota upon proteolytic fermentation of branched-chain amino acids have been less investigated, and data on their metabolic effects are limited. Whether these BCFAs show pro-/anti-inflammatory effects needs further exploration.

Fermented feed usage has showed beneficial effects on the gut microbiota of piglets. Yuan et al. suggested that fermented soybean meal using *Bacillus subtilis*, *Hansenula anomala* and *Lactobacillus casei* increased lactic acid bacterial counts and decreased fecal *Escherichia coli* counts in weaned piglets [10]. Additionally, dietary fermented soybean meal increased the relative abundance of *Bacteroidetes* and *Prevotellaceae_NK3B31_group* and decreased the relative abundance of *Proteobacteria* and *Actinobacillus* [22]. Consistent with these findings, our results showed that 10%, 50% and 100% fermented corn–soybean meal mixed feed induced an increase in the abundance of *Lactobacillus*. This bacterium, which is the main butyrate-producing bacterium in the colon, has been repeatedly reported to be crucial for the prevention of pathogen infection and alleviation of intestinal inflammation [33]. This observation might be in line with the results that 10%, 50% and 100% fermented mixed feed increased the levels of colonic butyrate. Furthermore, 100% fermented corn–soybean meal mixed feed also resulted in an increased abundance level of *Faecalibacterium*. *Faecalibacterium* has been shown to alleviate inflammation and has great potential for intestinal health enhancement [34]. Moreover, a previous study reported that the genus *Faecalibacterium* was positively correlated with feed efficiency and fatness traits in Duroc pigs [35]. Taken together, these findings indicate that 100% fermented corn–soybean meal increased the abundance of colonic *Lactobacillus* and *Faecalibacterium*, which might be associated with the reduced diarrhea incidence and intestinal inflammation and thus with the improvement in growth performance.

## 4. Materials and Methods

All animal experiments were carried out in accordance with the ARRIVE guidelines [36] and were approved by the Animal Care and Use Committee of the Institute of Animal Science, Guangdong Academy of Agricultural Sciences (authorization number GAASIAS-2017-012).

### 4.1. Preparation of Fermented Feed and Laboratory Analysis

The control diet was formulated to meet the 2012 nutrient recommendations of the National Research Council (NRC) [37] (Table 2). A total of 1000 kg of basal substrates containing 78.79% corn and 21.21% soybean meal was thoroughly mixed with 370 L of microbial suspensions containing yeast (4 × 10^7^ cfu/mL), *Bacillus subtilis* (4 × 10^7^ cfu/mL), Bacillus Licheniformis (4 × 10^7^ cfu/mL), *Lactobacillus plantarum* (2 × 10^7^ cfu/mL) and *Lactobacillus reuteri* (2 × 10^7^ cfu/mL). Then, the mixed substrates were transferred to a polythene bag equipped with a 1-way valve at room temperature (32–37 °C) for 4 days. After that, the fermented substrates with 37% moisture content were kept in an air-conditioned room (16 °C).

Both fermented substrates and non-fermented substrates (approximately 3 g) were mixed thoroughly with 10 mL of ultrapure water and then centrifuged at 5000× *g* for 15 min. The supernatant was collected to determine the pH value using an electronic pH meter (HI 8242C; Beijing Hanna Instruments Science & Technology, Beijing, China). According to the methods of the Association of Official Analytical Chemists (AOAC, 2008) [38], the contents of acid detergent fiber (AOAC Method 973.18), crude fat (AOAC Method 960.39) and crude protein (AOAC Method 992.15) in fermented and non-fermented substrates were measured and calculated on an 88% dry matter (AOAC Method 950.46) basis. The lactic acid contents were measured using a lactic acid enzymology assay kit (Nanjing-Jiancheng Bio Co., Nanjing, China) in accordance with the manufacturer’s protocol. The contents of anti-nutritional factors in diet feed before and after fermentation were determined with commercial kits (Nanjing Jiancheng Institute of Bioengineering, Nanjing, China) in accordance with the manufacturer’s instructions. Finally, moist fermented substrates (approximately 100 g) were used to measure the abundance of microorganisms.

### 4.2. Microbial Determinations

A total of 2 g of fermented feed at different incubation times (0, 1, 3, 6 and 9 d) was obtained and diluted (1:9 (*w*/*v*)) with sterile water. The suspension sample was gently and thoroughly vortexed for 5 min; then, ten-fold dilutions were prepared with sterile water. A volume of 0.1 mL of suspension liquid was taken and plated on selective media. *Lactobacillus* was counted on MRS agar following anaerobic incubation at 37 °C for 2 d. Yeasts were measured on yeast extract peptone dextrose agar with 50 mg/L chloramphenicol following aerobic incubation at 30 °C for 2 d. *Bacillus* abundance was measured in nutrient broth agar by morphological and biochemical identification after aerobic incubation at 37 °C for 1 d.

### 4.3. Animals and Experimental Design

A total of 128 weaned piglets Duroc×(Landrace×Yorkshire), 21-day-old barrows with an initial body weight of 5.75 ± 0.01 kg) were randomly allocated to 4 treatment groups for a 14 d feeding trial. Each treatment contained 8 replicate pens, with 4 piglets per pen. The piglets in the control group (CON) were fed the control diet. Piglets in the FMF-10, FMF-50 or FMF-100 group received a diet in which a portion of 10%, 50% or 100%, respectively, of the mixed substrates of corn and soybean meal was replaced with fermented substrates. The piglets were fed four times (at 8:00, 11:00, 15:00 and 18:30) per day and had free access to water throughout the 14 d experimental trial. Feed residue in each pen was collected and weighed daily, and feed intake in each pen was recorded every day for the calculation of ADFI. All piglets were weighed at the beginning and end of the experimental trial after overnight fasting (12 h) to obtain ADG and FCR. Fecal consistency for each pig in each pen was visually assessed twice (at 8:00 and 14:00) each day by two independent observers throughout the entire feeding trail. Fresh excreta were scored as follows: 0, normal; 1, pasty; 2, semi-liquid; and 3, liquid. Piglets with a daily fecal score of ≥2 were considered to be suffering from diarrhea. Diarrhea incidence was calculated as follows: Diarrhea incidence (%) = Total number of pigs per pen with diarrhea/(Number of pigs per pen × n) × 100, where n represents the experimental duration in days.

### 4.4. Determination of Apparent Total-Tract Nutrient Digestibility

A total of 12 weaned piglets (Duroc×(Landrace×Yorkshire), 21-day-old barrows with an initial body weight of 5.90 ± 0.22 kg) were randomly assigned to the CON and FMF-100 groups, and the feeding trial lasted 14 days. Each group contained 6 replicate pens, with one piglet per pen. On the 10th, 11th and 13th days of the experiment, fecal samples were collected into plastic containers at 8:00 am and 8:00 pm each day. Before chemical analysis, the collected fecal samples were dried at 65 °C for 72 h and then ground and filtered through a 1 mm sieve. Crude fat (AOAC Method 960.39), crude protein (AOAC Method 992.15) and crude fiber (AOAC Method 962.09) in the fecal samples were analyzed according to AOAC methods (2008) [38]. The gross energy of feces was measured using an adiabatic oxygen bomb calorimeter (Parr Instrument Co., Moline, IL, USA).

### 4.5. Sample Collection

At the end of the study, one pig from each pen was randomly selected, anesthetized with sodium pentobarbital (40 mg/kg body weight) and then sacrificed. The entire intestine was quickly taken out and put on a cold tray to obtain samples of the duodenum, jejunum, ileum and colon. Segments of approximately 2.0 cm in length of the duodenum, jejunum and ileum were selected, rinsed with cold phosphate buffer solution (PBS) and then fixed with 4% paraformaldehyde for histochemical staining and morphometric evaluation. Portions of approximately 20 cm of the duodenum, jejunum, ileum and colon were opened longitudinally, cleaned with cold PBS and scraped with sterile glass microscope slides to collect mucosal samples. The collected mucosal samples were snap-frozen in liquid nitrogen and stored at −80 °C for further analysis. The colon contents were also collected, frozen in liquid nitrogen and stored at −80 °C to analyze gut microbiota and short-chain fatty acids (SCFAs).

### 4.6. Intestinal Morphology Analysis

The fixed samples of duodenum, jejunum and ileum were embedded in paraffin, and 4 μm cross-sections from each specimen were mounted on slides coated with polylysine, deparaffinized, rehydrated and then stained with hematoxylin–eosin (HE) to evaluate the intestinal morphology. The stained slices were then scanned with a digital brightfield microscope scanner (Pannoramic 250; 3D HISTECH, Budapest, Hungary). Twenty-five well-oriented and intact villi and adjacent crypts from each sample were randomly selected to determine villus height and crypt depth in each segment using slide viewer software (Case Viewer 2.3; 3D HISTECH, Hungary), and the villus height-to-crypt depth ratio (VCR) was calculated.

### 4.7. Quantitative Real-Time PCR (qPCR)

mRNA from the duodenal, jejunal, ileal and colonic mucosal samples was extracted with TRIzol reagent (Invitrogen, Carlsbad, CA, USA). mRNA concentration and quality were assessed with a NanoDrop-ND1000 spectrophotometer (Thermo Fisher Scientific Inc., Walldorf, Germany). A total of 1 μg of RNA of each sample was reverse-transcribed into cDNA with a PrimeScript™II 1st Strand cDNA Synthesis Kit (Takara, Tokyo, Japan). Ten-fold diluted cDNA, SYBR green I (Bio-rad, Hercules, CA, USA) and gene-specific primers (Table 3) in a final volume of 20 μL were used to perform qPCR analysis in triplicate. The fold change in the expression of the target genes in piglets fed FMF-10, FMF-50 and FMF-100 diets (treatment groups) was normalized to *ACTB* and relative to the expression of those in piglets fed the control diet (as “1”) and was calculated for each sample using the 2^−ΔΔCt^ method [39], where ΔΔCt = (Ct, Target − Ct, *ACTB*)_Treatment group_ − (Average Ct, Target − Average Ct, *ACTB*)_Control group_.

### 4.8. Western Blot Analysis

Approximately 0.5 g of frozen duodenal, jejunal or ileal mucosa was used to extract protein with 1 mL of RIPA buffer containing 1% protease inhibitor cocktail and 1% phosphatase inhibitor at 4 °C for 30 min; then, the mixture was centrifuged at 12,000× *g* at 4 °C for 15 min. A BCA protein assay kit (Pierce, Rockford, IL, USA) was used to determine protein concentration according to the manufacturer’s protocol. Denatured proteins were separated on 8–10% SDS-PAGE gel and then transferred to nitrocellulose membranes. The membranes were blocked with 5% BSA in TBST buffer for 30 min at room temperature. After having been washed 4 times, the membranes were incubated with diluted primary antibodies at 4 °C overnight. They were then washed 4 times and incubated with the HRP-labeled secondary antibodies for 1 h at room temperature. After another 4 washes, we visualized immunoreactive proteins using Immobilon Western Chemiluminescent HRP Substrate (Millipore, Billerica, MA, USA) and the VersaDoc Imaging System (Bio-Rad, Hercules, CA, USA), and ImageJ software version 2 (National Institutes of Health, Bethesda, MD, USA) was used to quantify protein band intensity. Primary antibodies against ZO-1 (D6L1E), occludin (E6B4R), Claudin1 (D5H1D) and β-actin (13E5) were obtained from Cell Signaling Technology (Boston, MA, USA), and the dilution for these primary antibodies was 1:1000. The results are shown as the abundance of each target protein relative to β-actin.

### 4.9. Measurement of pH Value in the Stomach, Duodenum, Jejunum, Ileum and Colon, and Colonic SCFA Concentration Analysis

Digesta from the stomach, duodenum, jejunum, ileum or colon were collected and mixed in a Ziplock bag; then, the pH value of the prepared digesta samples was measured thrice using a portable HI 9024C pH meter (HI 8242C; Beijing Hanna Instruments Science & Technology, Beijing, China). The levels of SCFAs including acetate, propionate, butyrate, valerate, isobutyrate and isovalerate in the colon contents were analyzed using gas chromatography–mass spectrometry (GC-MS) in accordance with a previous study [40]. Briefly, approximately 0.05 g of colon content was mixed with 1 mL of 25% metaphosphoric acid and vortexed thoroughly for 15 min. The homogenized mixture was then extracted with 1 mL of diethyl ether for 10 min and subsequently centrifuged at 12,000× *g* for 10 min at 4 °C. The supernatant was collected and filtered through a 0.22 μm filter before being subjected to GC-MS. SCFA contents were determined with the Agilent 7890B-7000D GC/MS system and the HP-INNOWAX (25 m × 0.20 mm, 0.40 μm) column. The column temperature was initially held at 100 °C for 5 min and then increased to 150 °C at 5 °C/min and finally to 240 °C at 30 °C/min and held at 240 °C for 2 min. The detector was run in electron impact ionization mode (electron energy of 70 eV).

### 4.10. Gut microbiota Determination

DNA extraction and 16S rDNA sequencing: Microbial genomic DNA from colonic contents was extracted by using the CTAB method. Agarose gel electrophoresis was used to monitor the purity and concentration of DNA. Bacterial DNA was diluted to 1 ng/μL for sequencing library preparation. The diluted DNA was used as a template, Phusion^®^ High-Fidelity PCR Master Mix with GC Buffer (New England Biolabs, Ipswich, MA, USA) was used as the reaction system, and the primer pair (forward primer, 515F: 5′-GTGCCAGCMGCCGCGG-3′; reverse primer, 806R: 5′-GGACTACNVGGGTWTCTAA-3′) with attached barcode sequences was used to amplify the V4 hypervariable region of the 16S rRNA gene. The PCR amplicon products were purified using 2% agarose gels and were recycled using a GeneJET Kit (Thermo Scientific, Waltham, MA, USA). The libraries of amplicons were constructed using Ion Plus Fragment Library Kit 48 rxns (Thermo Fisher, Waltham, MA, USA) and quality-controlled on Qubit (Life Technologies, Carlsbad, CA, USA). The pooled libraries were sequenced on the Thermo FisherIon S5TM XL platform (Novogene, Beijing, China).

Sequence bioinformatics analysis: All raw reads were cut into low-quality bases by using Cutadapt (V1.9.1) and assigned to each sample according to the barcode sequences; then, the chimeric sequences were filtered out by adopting the UCHIME algorithm to obtain clean reads. Then, Uparse (v7.0.1001) was applied to cluster the clean reads to obtain the operational taxonomic units (OTUs) based on 97% sequence similarity. Representative sequences of OTUs were selected according to the maximum frequency, and OTUs with fewer than two clustered sequences were filtered out. Next, all representative OTUs were aligned to the SILVA database SSUrRNA by using Mothur software version 1.40.45 at a threshold of 0.8~1 to obtain the taxonomic classification (from phylum to genus). The phylogenetic tree of OTUs was built using MUSCLE (V3.8.31), and the uniformization processing of the samples was conducted based on the lowest number of data to obtain OTU abundance for downstream analysis. Alpha diversity indices (including observed species, Chao1, Shannon, Simpson, Ace and PD_whole_tree) and beta diversity indices (unweighted and weighted UniFrac distance) were calculated in QIIME (V1.9.1).

### 4.11. Statistical Analysis

The data were analyzed using the IBM SPSS statistics V26.0.0 software package (IBM Corp., Armonk, NY, USA). The Shapiro–Wilk test was used to evaluate all variables that showed a normal or non-normal distribution. All normally distributed data were analyzed by 1-way ANOVA followed by Tukey’s post hoc test. Diet was the fixed effect, and pen was the random effect in the statistical model. Data are presented as means ± SEMs. Differences were considered significant if *p <* 0.05, and post hoc testing among treatments was conducted using Tukey’s multiple comparison tests. The data of apparent total-tract digestibility were analyzed by an unpaired *t*-test using GraphPad Prism version 8.0, and differences were considered significant if *p <* 0.05.

## 5. Conclusions

In conclusion, the results of the present study show that dietary 100% fermented corn–soybean meal supplementation increased ADG and ADFI and decreased diarrhea incidence in weaned piglets. Fermented corn–soybean meal supplementation also improved the intestinal morphology, increased tight junction protein expression and decreased intestinal inflammation. Additionally, 100% fermented corn–soybean meal supplementation selectively increased colonic butyrate levels and the abundance of *Faecalibacterium* and *Lactobacillus* and reduced the concentration of BCFAs. Although no significant effect of 10% or 50% fermented corn–soybean meal supplementation on growth performance and diarrhea incidence in weaned piglets was found, these two fermented corn–soybean meal diets exhibited an anti-inflammatory effect by increasing the abundance of *Lactobacillus* and the levels of propionate and butyrate in the colon. Taken together, this study suggests that fermented corn–soybean meal feed has great potential for improving growth performance and gut health in weaned piglets.

## Figures and Tables

**Table 1:** Effects of fermented corn–soybean meal feed on nutrient apparent digestibility in weaned piglets ^1^.

Item	CON	FMF-100	*p*-Value
Apparent total-tract digestibility of gross energy (%)	90.25 ± 0.41	91.19 ± 0.39	0.13
Crude protein digestibility (%)	97.81 ± 0.07	98.28 ± 0.06	<0.01
Crude fat digestibility (%)	87.78 ± 1.15	88.28 ± 1.31	0.78
Crude fiber digestibility (%)	66.54 ± 6.07	73.90 ± 5.17	0.04

^1^ Values are means ± SEMs; *n* = 6 per treatment. Differences were considered significant if *p <* 0.05. CON, control diet; FMF-100, control diet supplemented with 100% fermented corn–soybean meal mixed feed (FMF).

**Table 2:** Ingredient composition and nutrient levels in control diet (%, air-dried basis).

Item	CON	FMF-10	FMF-50	FMF-100
Ingredient composition, %				
Corn	52	46.8	26	0
Soybean meal	14	12.6	7	0
Fermented corn–soybean meal mixed feed	0	6.60	33.00	66.00
Soybean hull	3.64	3.64	3.64	3.64
Whey protein concentrate	6	6	6	6
Low-protein whey powder	10	10	10	10
Fishmeal	4	4	4	4
Soybean oil	3	3	3	3
Sucrose	0.9	0.9	0.9	0.9
Lactose	2.1	2.1	2.1	2.1
Calcium citrate	0.3	0.3	0.3	0.3
Calcium hydrogen phosphate	1.8	1.8	1.8	1.8
L-Lys-HCL	0.7	0.7	0.7	0.7
DL-Met	0.3	0.3	0.3	0.3
L-Thr	0.34	0.34	0.34	0.34
L-Trp	0.06	0.06	0.06	0.06
Sodium chloride	0.2	0.2	0.2	0.2
Choline chloride 50%	0.2	0.2	0.2	0.2
ZnO	0.2	0.2	0.2	0.2
^1^ Vitamin–mineral premix	0.26	0.26	0.26	0.26
Total	100	100	100	100
^2^ Calculated nutrient levels (88% dry matter basis)				
DE, kcal/kg	3552	3552	3552	3552
Ca, %	0.79	0.79	0.79	0.79
CP, %	19.77	19.77	19.77	19.77
SID Lys, %	1.59	1.59	1.59	1.59
SID Met, %	0.585	0.585	0.585	0.585
SID Thr, %	1.023	1.023	1.023	1.023
SID Trp, %	0.27	0.27	0.27	0.27
Available P, %	0.55	0.55	0.55	0.55
Total P, %	0.73	0.73	0.73	0.73

^1^ Supplied per kilogram of complete diet: Fe, 120 mg; Cu, 10 mg; Zn, 120 mg; Mn, 35 mg; I, 0.25 mg; Se, 0.2 mg; calcium pantothenate, 10 mg; all-trans retinol equivalent, 8000 IU; cholecalciferol equivalent, 1000 IU; RRR-α-tocopherol equivalent, 110 mg; phylloquinone equivalent, 2 mg; thiamine equivalent, 2 mg; riboflavin equivalent, 6 mg; pyridoxine equivalent, 4.0 mg; cyanocobalamin equivalent, 0.02 mg; nicotinamide equivalent, 25 mg; folic acid, 1.0 mg; biotin, 0.25 mg. ^2^ Nutrient levels were calculated based on the NRC (2012) database. CON, control group, piglets fed a control diet; CP, crude protein; DE, digestible energy; SID, standardized ileal digestibility; FMF-10, FMF-50 and FMF-100, control diet supplemented with 10%, 50% and 100% fermented corn and soybean mixed feed (FMF), respectively.

**Table 3:** Primer sequences used in this study.

Genes	Sequences (5′–3′)		Product Size (bp)	GenBank Accession Number
*TNF*	Forward Reverse	CACGCTCTTCTGCCTACTGC GTCCCTCGGCTTTGACATT	164	NM_214022.1
*ACTB*	Forward Reverse	CATCGTCCACCGCAAAT TGTCACCTTCACCGTTCC	210	NC_010445
CLDN1	Forward Reverse	GATTTACTCCTACGCTGGTGAC CACAAAGATGGCTATTAGTCCC	199	NM_001244539.1
OCLN	Forward Reverse	GCACCCAGCAACGACAT CATAGACAGAATCCGAATCAC	144	XM_005672525
*IL10*	Forward Reverse	GAAGCGCATCGAGGCCATTC AGGCACTCTTCACCTCCTC	162	NM_214015.1
*IL1B*	Forward Reverse	CTCCAGCCAGTCTTCATTGTTC TGCCTGATGCTCTTGTTCCA	230	NM_214055.1
*TJP1*	Forward Reverse	AGCCCGAGGCGTGTTT AGCCCGAGGCGTGTTT	147	XM_021098827.1

*ACTB*, actin beta; *IL1B*, Interleukin 1 beta, *IL10*, interleukin 10; *TNF*, tumor necrosis factor alpha; *OCLN*, *occludin*; *TJP1*, tight junction protein 1.

## Data Availability

Data is contained within the article.
